# Working conditions, job stress and work-related consequences among hospital employees—differences by professional group, working hours and job levels: A cross-sectional study

**DOI:** 10.1371/journal.pone.0343567

**Published:** 2026-03-12

**Authors:** Nicole R. Hander, Rebecca Erschens, Thomas Klein, Marc N. Jarczok, Nadine Mulfinger, Monika A. Rieger, Florian Junne, Peter Angerer, Bernd Puschner, Andreas Müller, Janna Küllenberg, Imad Maatouk, Stefan Süß, Elena Gesang, Sascha Ruhle, Harald Gündel, Eva Rothermund-Nassir

**Affiliations:** 1 Department of Psychosomatic Medicine and Psychotherapy, Ulm University Medical Center, Ulm, Germany; 2 Department of Psychosomatic Medicine and Psychotherapy, Internal Medicine, University Medical Hospital Tübingen, Tübingen, Germany; 3 Institute of Occupational and Social Medicine and Health Services Research, University Hospital Tübingen, Tübingen, Germany; 4 Department of Psychosomatic Medicine and Psychotherapy, Otto-von Guericke-University of Magdeburg, Magdeburg, Germany; 5 Institute of Occupational, Social and Environmental Medicine, chs, Medical Faculty, Heinrich-Heine-University Düsseldorf, Düsseldorf, Germany; 6 Department of Psychiatry II, Ulm University and Bezirkskrankenhaus Günzburg, Günzburg, Germany; 7 Institute of Psychology, Work and Organisational Psychology, University of Duisburg-Essen, Duisburg-Essen, Germany; 8 Institute for Medical Psychology, Centre for Psychosocial Medicine, University Heidelberg, University Hospital Heidelberg, Heidelberg, Germany; 9 Department for General Internal Medicine and Psychosomatics, University Hospital Heidelberg, Heidelberg, Germany; 10 Chair of Business Administration, in particular Work, Human Resource Management and Organisation Studies, Heinrich-Heine-University Düsseldorf, Düsseldorf, Germany; University of Trieste: Universita degli Studi di Trieste, ITALY

## Abstract

**Objective:**

Hospital employees face significant occupational stress that negatively impacts both their well-being and organizational outcomes. This challenge is amplified during times of staff shortages, economic difficulties and conflicting roles. To support hospital workers effectively and create a positive work environment, it is crucial to identify specific groups experiencing greater challenges. This study examines how working conditions, job stress, and related consequences vary across professional groups, working hours, and job levels within hospitals.

**Methods:**

The study analyzed data from 406 employees (66% female) across three German hospitals, collected between December 2019 and January 2020 as part of the SEEGEN (Mental Health at the Hospital Workplace) study on mental health in hospital workplaces. Group differences in target variables were investigated via between-group one-way independent analyses of variance.

**Results:**

Results showed that nurses, part-time employees, and non-leadership staff reported the most significant needs for improvement. Nurses experienced the lowest effort-reward balance, reduced job satisfaction, and the highest intention to leave their jobs. Part-time workers felt less control over their work decisions, perceived poorer cooperation among occupational groups, and reported a weaker psychosocial safety climate compared to full-time employees. They were also more irritated, less satisfied, and more inclined to consider leaving their jobs. Additionally, part-time workers rated their employers as less attractive and were less likely to recommend them. Leadership positions appeared to offer a protective effect against some of these negative outcomes.

**Conclusions:**

In conclusion, this study provides a comprehensive view of the differing work stressors and consequences faced by hospital staff based on their roles, work hours, and job levels. These insights emphasize the importance of tailoring interventions to target specific groups within hospitals to enhance occupational health and create supportive work environments.

**Trial registration:**

German Clinical Trial Register (DRKS): DRKS-ID DRKS00017249 (Registration Date: 8^th^ October 2019).

## Background

Hospital employees face massive occupational stress [1–3] due to challenging working conditions [4–8]. These circumstances are related to negative work-related consequences at the individual level, such as reduced job satisfaction [9,10], and at the organizational level, such as increased intention to leave [11]. These challenges are well known [12], especially in the nursing field, and are becoming increasingly relevant in times of a shortage of a skilled workforce, pressure to economize, role conflicts and work intensification and complexity [11,13]. Owing to demographic changes and the lack of healthcare professionals [14], health services face the challenge of having to meet increasing demands with limited resources.

Working conditions, employees’ job stress, and work-related consequences for the individual and the organization are interrelated and form a critical foundation for institutional resilience and performance [15]. Previous research has shed some light on differences in professional groups, working hours, and job levels among groups of hospital workers.

### Professional group

With respect to working conditions across different professional groups, physicians indicated greater demands, more influence at work, more possibilities for development and more social support than did nurses in emergency medicine in Serbia [16]. In Swiss acute care and rehabilitation hospitals, nurses reported greater job demands and lower well-being, physicians showed highest quantitative demands and work–life conflicts, and medical-technical professionals experienced more job insecurity and less influence at work compared to other health professionals [17]. Data from a staff survey at two German university hospitals revealed that work-related variables such as influence and degree of freedom at work, possibilities for development, meaning of work, workplace commitment, role conflicts, social relations, job satisfaction, and patient-related burnout were significantly more positively assessed by physicians than by nurses [5]. Conversely, nurses perceived more positive collaborative relationships than did physicians or other healthcare professionals in Germany and Taiwan [5,18]. With respect to job satisfaction across professional groups, previous studies have shown that physicians generally reported greater job satisfaction than did nurses or other staff [5,18–20]. In a Finnish university hospital, physicians constituted the most satisfied group, nurses and maintenance staff were the least satisfied, and office and administrative staff were fairly satisfied [19]. An analysis in a German university pediatric hospital indicated that only every fourth employee and only every tenth physician were satisfied with their working conditions [21]. Satisfaction with pay was significantly greater among physicians than among nurses in Germany [22].

### Working hours

Previous studies revealed disparities between part- and full-time workers in medical practices [23,24], but studies in hospitals are scarce. One study conducted in Germany indicated that full-time physicians exhibited higher levels of work-family-conflict compared to part-time physicians working either in hospital or in ambulatory care [25]. Results of general internists in Switzerland showed that part-time compared to full-time work was associated with a lower risk of poor well-being [26].

### Job level

With respect to job levels, a large-scale study in European hospitals confirmed that clinical leaders perceived teamwork and safety climates more positively than frontline clinicians did [27]. Leadership–followership dynamics entail inherent asymmetries in status, expertise, identity, and power. Such imbalances frequently limit followers’ autonomy [28], which, as outlined in the job-demand-control-support model, is associated with increased job strain [29]. These findings might explain why assistant doctors in German hospitals had a significantly greater prevalence of distress than specialists or chief physicians [30]. Additionally, these findings shed light on why individuals in both lower management positions and those without management responsibilities experience work stressors and their long-term consequences to a greater extent [31].

Taken together, many studies have mainly focused on one or two groups in hospitals, such as nurses or physicians [9,32,33]. Therefore, interventions are often developed for one group without considering the needs of the other groups within a holistic framework [32]. For a comprehensive understanding of how hospital employees cope with stress and to identify areas where structural improvements are necessary, it is important to examine group differences in stressors, stress reactions, and outcomes. However, these topics have been predominantly studied independently of each other. Only one study pursued this approach and found promising results on the extent of work stressors, stress reactions and long-term consequences among different professional groups in Swiss acute care and rehabilitation hospitals [17].

To address this gap in knowledge, the present exploratory analysis aims to describe and compare working conditions, stress experiences, and job-related outcomes among different professional groups, working time models, and hierarchical levels within hospitals, and to explore potential patterns that may inform future research. Specifically, it evaluates working conditions, perceived effort-reward imbalance, cognitive and emotional strain, and job satisfaction, aiming to detect potential differences. With respect to organizations, it investigates perceptions related to employees’ intention to leave, employer attractiveness, and willingness to recommend the workplace to others.

## Methods

### Study design

This cross-sectional study used baseline data from a cluster-randomized trial called “Mental health in the hospital workplace” (in German: SEElische Gesundheit am Arbeitsplatz KrankeNhaus – SEEGEN). This study was conducted according to the guidelines of the Declaration of Helsinki and approved by the institutional review boards of Ulm University (501/18), Heinrich-Heine University Düsseldorf (6193R), and Heidelberg University (S-602/2019). The investigation was registered by the German Clinical Trial Register (DRKS) under ID number DRKS00017249 (Registration Date: October 8th, 2019). The SEEGEN trial evaluated the effects of a complex intervention on the mental health and well-being of hospital employees [34]. The study took place in three hospitals located in Germany (A: owned by a private health company; B: community hospital; C: university hospital). The results were reported in line with the STROBE statement. Detailed study information has been published [35].

### Sample and procedures

Potentially eligible participants were employees between 18 and 70 years of age at the three study sites working in medical, nursing, functional or other services. The selected age range corresponds to the usual employment period in the German healthcare sector. The exclusion criterion was a lack of sufficient German language skills ensuring accurate comprehension of self-reported measures in a questionnaire study. All participants provided written informed consent.

Baseline data were collected between 01 December 2019 and 31 January 2020 by convenience sampling. These data represent the baseline assessment (T0) of a randomized controlled trial with two additional measurement points (June-July 2020 and December 2020-January 2021) evaluating a psychosocial intervention [34]. Through posters and announcements from supervisors, hospital employees were invited to information events. Additionally, study personnel directly approached potential trial participants in meetings at all participating wards. The questionnaires were completed on paper or online. The participants were encouraged to answer the questions in private and as spontaneously as possible.

The overall target sample size was determined for the evaluation of the intervention effect, based on the primary outcome (change in total Irritation Score from baseline to post-intervention). Assuming a medium effect size (d = 0.4), α = 0.05, and power = 0.80, the planned total sample was 720 participants to allow for expected attrition [35]. The final sample size was smaller than initially planned due to challenges with recruitment and retention during the SARS-CoV-2 pandemic.

### Measures

Sociodemographic characteristics were assessed using a self-developed questionnaire comprising the following items: gender (male/female/diverse), age (continuous), marital status (married/single/divorced/widowed), employment status (full-time/part-time/leave/other), professional group (medical/medical-technical/nursing/secretarial staff/functional service/other), shift-working (yes/no), leading position (yes/no), hierarchy level (top management/middle management/no management), years of professional experience (continuous). Full details are provided in Supporting File 1 (S1 File). For reasons of anonymization, data from the ‘male’ and ‘diverse’ categories were aggregated. All outcomes were measured via standardized self-rated scales with established psychometric properties.

Three subscales of the German questionnaire for hospital employees, “Screening Work Analysis Instrument for Hospitals-Self Report Version” (TAA-KH-S; [36]), were used to measure working conditions: (1) Job decision authority (shortened from nine to three items), (2) quantitative job demands (three items) and (3) cooperation between occupational groups (shortened from five to two items). Items were rated on a five-point Likert scale ranging from 1 (no, not at all) to 5 (yes, absolutely), with a higher mean of the raw scores reflecting greater job decision authority/more quantitative job demands/greater cooperation between occupational groups. In the present sample, internal consistency for the three scales was good (Cronbach’s α = .72 to.87), which is in line with previous validation studies reporting α between .68 and .82 and supporting content and construct validity [36,37]. The instrument has been repeatedly used in German speaking countries, and is especially adapted to health-care professions [38].

The Psychosocial Safety Climate (PSC-12; [39]) measures an organizational climate that consists of four dimensions: organization participation, organization communication, management priority and management commitment. The items such as “Psychological well-being of staff is a priority for this organization.” are rated on a five-point Likert scale ranging from 1 (strongly disagree) to 5 (strongly agree). The higher the sum score is, the better the psychosocial safety climate. According to the benchmark risk level [40], scores ≤ 26 are associated with very high preeminent psychosocial risk factors at work for impaired mental health, and scores between < 27 and ≤ 37 are associated with high risk. Cronbach’s alpha was 0.94 at T0, indicating excellent reliability, which aligns with prior studies [41]. Evidence for the PSC scale’s validity includes confirmatory factor analyses supporting its dimensional structure and convergent correlations with outcomes like burnout and engagement [42].

Perceived emotional and cognitive strain in the context of the working environment was measured by the Irritation Scale [43]. It consists of eight items, such as “I have difficulty relaxing after work.”, rated on a seven-point Likert scale ranging from 1 (strongly disagree) to 7 (strongly agree). The higher the mean score is, the greater the degree of irritation. The Irritation Scale demonstrated excellent reliability (α = 0.87), with validity and reliability confirmed across 15 studies in different occupational sectors [43].

The short version of the Effort-Reward-Imbalance (ERI) scale captures the relationship between effort spent and rewards received [44]. Rewards are not restricted to wages, e.g., “Considering all my efforts and achievements, I receive the respect and prestige I deserve in my work”. This short version for self-assessment of employees consists of three items measuring effort and seven items measuring reward, each on a four-point Likert scale from 1 (strongly disagree) to 4 (strongly agree). The Effort-Reward (ER) ratio is calculated by dividing the total effort score by the total reward score, with values < 1 indicating less effort for each reward and values > 1 indicating more effort for each reward. The ERI scale showed good reliability (Effort: α = .69; Reward: α = 76) similar to other studies [44], with confirmed factorial and convergent validity.

Job satisfaction was assessed with the short version of “The General Job Satisfaction Scale” [45,46]. The eight items, e.g., “I really enjoy my work” and “Are you satisfied with your opportunities for advancement?”, are measured on five-point Likert scales. The higher the sum score is, the greater the degree of job satisfaction. It showed good reliability (α = 0.77), and its short 8-item version is nearly identical in psychometric quality to the longer scale.

Intention to leave was measured by the item “How often during the past twelve months have you thought about quitting your profession?” from the standard German version of the COPSOQ [47] and of the European NEXT study [48] on a five-point scale (“never“, “a few times a year”, “a few times a month”, “a few times a week”, “every day”), with established psychometric properties.

Employer attractiveness was assessed by five items, e.g., “I enjoy working for my employer” and “I feel emotionally attached to my employer” measured on a seven-point Likert scale from 1 (strongly disagree) to 7 (strongly agree) [49]. The higher the sum score is, the greater the degree of employer attractiveness. The Employer Attractiveness Scale demonstrated very good reliability (Cronbach’s α = 0.89), consistent with a prior study [49], alongside established construct reliability and validity.

Recommendation of the employer was measured by the item “How likely is that you would recommend this company to a friend or colleague?” on an eleven-point Likert scale ranging from 1 (highly unlikely) to 11 (highly likely) (modified version of [50,51]) which is a widely used single-item measure with established validity.

### Data analysis

Missing single items were replaced by the individual’s mean of the available values with the number of allowed missing values complying with the instructions of the test authors. To evaluate missing completely at random (MCAR) data for multivariate data with missing values, the test according to Little was used. A nonsignificant result indicates MCAR resulting in pairwise deletion.

The participants were considered managers if they held a top or middle management position. While a top management position refers to having ultimate responsibility for an entire unit, a middle management position is related to leadership responsibilities for subunits, such as senior physicians, nursing managers or team leaders. In the case of missing information at the hierarchy level, the variable “leading position” was considered and counted as leadership responsibility in the case of the answer “yes”. There were no participants with missing hierarchy levels or leading positions. For reasons of anonymization, professional groups were defined as “physicians”, “nurses” and “others”. Functioning services, medical-technical staff and administrative staff were considered as “others”. Additionally, employees were categorized as either “full-time workers” or “part-time workers.”

**Fig 1:**
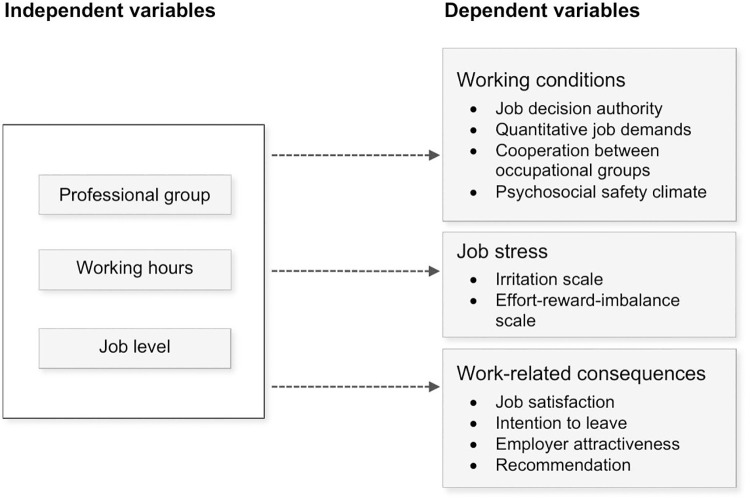
Overview of the studies’ independent and dependent variables.

Group differences in all target variables at the individual level were investigated via one-way independent ANOVAs (analysis of variance). Fig 1 provides an overview of the studies’ independent and dependent variables. P-values ≤ .05 were considered statistically significant. As this is an explorative study, significance testing was conducted to discover tendencies and not for confirmatory purposes. Thus, no adjustment for multiple testing was applied. The amount of clustering was measured by calculating the intraclass correlation coefficients (ICCs). When the ICCs were greater than.05 for the three participating hospitals, “hospital” was added as an additional between-subject factor (two-way independent ANOVA) to verify whether the one-way-independent ANOVA results would remain the same. However, for privacy reasons, no hospital-specific evaluations could be made. If the Levene test was significant, the more robust Welch ANOVA was used. With the between-group variable “professional group” containing more than two groups, post hoc tests were used. If equal sample sizes and homogeneity of variance were met, Tukey’s honestly significant difference (HSD) test would have been used. With different sample sizes, Hochberg’s GT2 was used. In the case of any violation of homogeneity of variance, the Games-Howell procedure would have been used in line with common practice [52].

To appraise clinical relevance, effect sizes were calculated. We reported the amount of variance in outcomes that was explained by the between-group variable (partial eta square, ɳ_p_^2^). In line with Cohen, we regarded ɳ_p_^2^ = 0.01 as small, ɳ_p_^2^ = 0.03 as medium and ɳ_p_^2^ = 0.14 as large effects. Furthermore, Cohen’s d = 0.3 represents a small effect, d = 0.5 a medium effect and d = 0.8 a large effect.

All data management and statistical analyses were conducted via IBM SPSS statistics 29 (IBM Corporation, Armonk, NY, USA).

## Results

### Demographics and baseline characteristics

**Fig 2:**
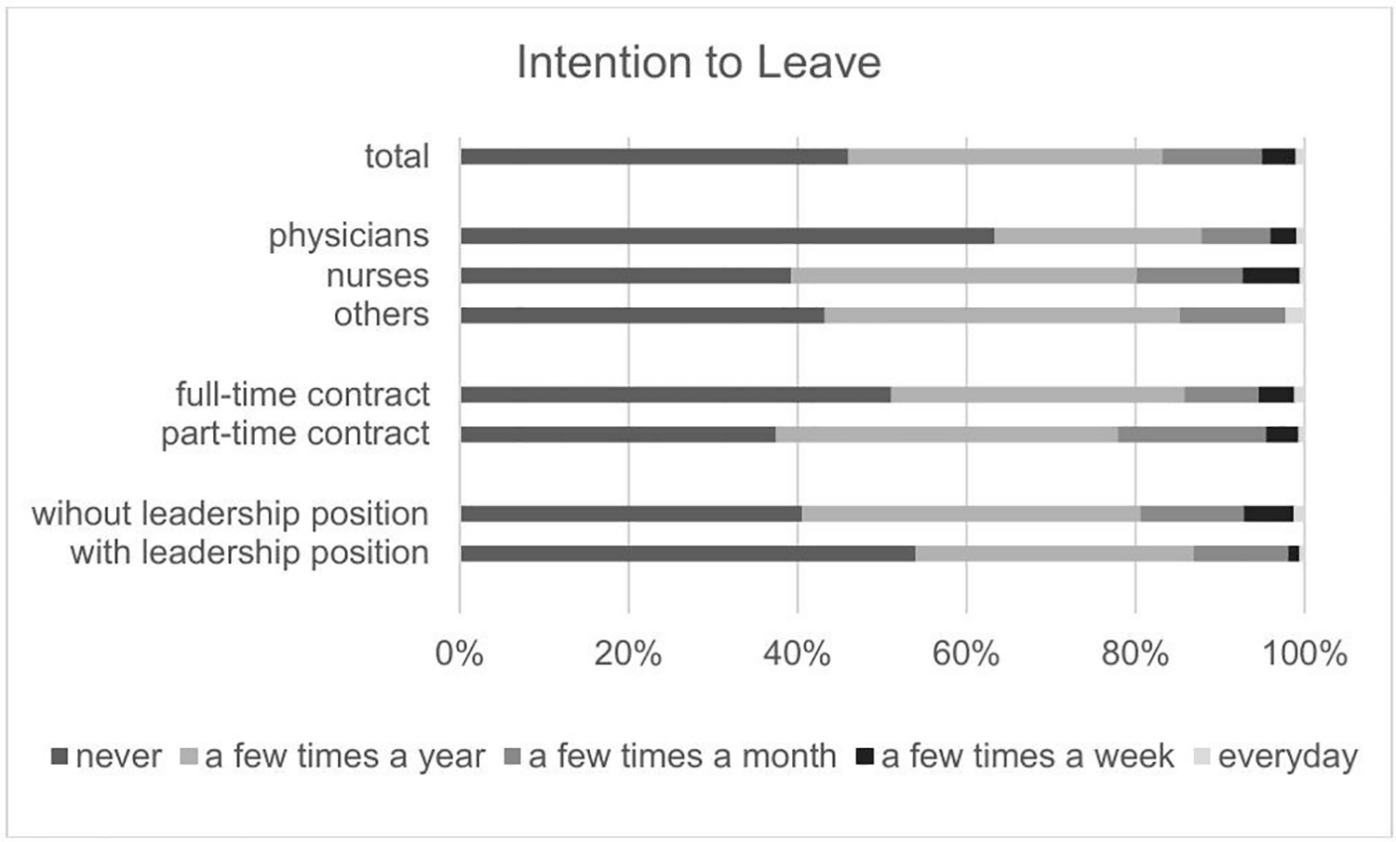
Intention to Leave across different groups of employees.

Of the N = 5654 potentially eligible hospital employees, N = 462 gave informed consent for their participation in the intervention study (8.2%). At baseline (T0), 406 study participants completed questionnaires (87.9%). The demographics of our sample and the study variables are displayed in Table 1 and Table 2. Fig 2 shows the variable “intention to leave” across different groups of employees. The sample consisted of 192 nurses, 110 physicians and 92 persons belonging to the functioning and other services, such as administration. While 262 workers were employed on a full-time basis, 140 were employed on a part-time basis. A total of 162 study participants held management positions, while 244 did not have management responsibilities. The nonsignificant result of Little’s missing completely at random test (χ2 = 122.496, df = 116, p = .322) indicated that the data were not systematically missing. There were 0% missing data present in the irritation scale, 1.5% in the PSC-12, 6.4% in job satisfaction, 7.9% in intention to leave, 11.1% in the ERI, 7.6% in job decision authority, 6.4% in quantitative job demands, 6.4% in cooperation between occupational groups, 6.4% in employer attractiveness and 6.9% in recommendations. The ICCs concerning hospitals were smaller than.05 for all variables except for PSC-12, employer attractiveness and recommendation.

**Table 1:** Characteristics of the study participants.

Sociodemographic data, n = 406	n	%
Gender (female)^1^	303	65.6
Age (years)		
21-30	70	17.8
31-40	91	23.2
41-50	93	23.7
50+	139	35.4
Missing	13	
Marital status (married/in partnership)^2^	291	73.1
Employment (full-time contract)^3^	262	65.2
Professional group		
Nursing Service	192	48.6
Medical Service	110	27.8
Functioning Service + other Function (e.g., Secretariats)	92	23.5
Missing	12	
Shift working (yes)^4^	218	54.5
Management responsibility (yes)^5^	162	39.9
Professional experience (years)		
0-10	88	22.8
11-15	45	11.7
16-25	85	22.0
26-30	48	12.4
31-35	56	14.5
36+	64	16.6
Missing	20	
Hospital setting		
Private	108	26.6
Community	169	41.6
University	129	31.8

^1^Missings: 0, ^2^Missings: 8, ^3^Missings: 4, ^4^Missings: 6, ^5^Missings: 0.

**Table 2:** Means and standard deviations for outcome variables (n = 406).

Outcome variables, N = 406	Mean	*SD*
Job decision authority^1^	3.25	0.91
Quantitative job demands^2^	3.52	0.87
Cooperation between occupational groups^3^	3.65	0.78
Psychosocial Safety Climate^4^	29.12	9.71
Irritation Scale^5^	26.15	9.54
Effort-Reward Imbalance Scale^6^	1.51	0.34
Job Satisfaction^7^	30.28	4.85
Intention to Leave^8^	1.77	0.88
Employer Attractiveness^9^	22.70	6.64
Recommendation^10^	7.08	2.76

^1^Missings: 31, [37], ^2^Missings: 26, [37], ^3^Missings: 26, [37], ^4^Missings: 6, [39], ^5^Missings: 0, [43], ^6^Missings: 45, [44], ^7^Missings: 26, [45], ^8^Missings: 32, [48], ^9^Missings: 26, [49], ^10^Missings: 28, [50]. Numbers in brackets refer to the corresponding sources in the reference list.

### Differences between professional groups

When analyzing differences between professional groups, five of the examined scales showed statistically significant variations: cooperation between occupational groups, psychosocial safety climate, effort-reward imbalance, job satisfaction, and intention to leave. (Table 3, Table 4). Results did not change when considering hospital as an additional factor. The mean score of cooperation was significantly higher for physicians than for nurses, with a small to medium effect. Nurses and others perceived a greater psychosocial safety climate than did physicians, with effect sizes in the moderate range. Although values were high for all groups, nurses reported a significantly greater effort-reward imbalance than did physicians or others, with small to medium effects. Compared with nurses, physicians perceived more job satisfaction and less intention to leave. Both differences represented medium effects.

**Table 3:** Working conditions, job stress and work-related consequences: comparisons between professional groups. ANOVAs with effect sizes.

	Physicians		Nurses		Others		Group effect		
Outcomes	n	Mean (SD)	n	Mean (SD)	n	Mean (SD)	F (df)	ɳ_p_^2^	p
Job decision authority^1^	99	3.40 (0.96)	177	3.16 (0.83)	87	3.20 (0.96)	(2, 185) = 2.33	0.007	.292
Quantitative job demands^2^	101	3.56 (0.91)	178	3.56 (0.87)	89	3.39 (0.80)	(2, 365) = 1.24	0.007	.292
Cooperation between occupational groups^3^	101	3.82 (0.73)	178	3.57 (0.80)	89	3.60 (0.81)	(2, 365) = 3.48	0.019	.032*
Psychosocial Safety Climate^4^	109	26.75 (8.65)	191	29.98 (10.31)	89	30.24 (9.01)	(2, 386) = 4.70	0.024	.010*
Irritation Scale^5^	110	26.28 (9.41)	192	25.48 (9.89)	92	27.28 (8.89)	(2, 391) = 1.13	0.006	.323
Effort-Reward Imbalance Scale^6^	98	1.44 (0.32)	171	1.57 (0.33)	81	1.46 (0.36)	(2, 347) = 5.44	0.030	.005*
Job Satisfaction^7^	101	31.51 (4.71)	178	29.54 (4.96)	89	30.42 (4.73)	(2, 365) = 5.37	0.029	.005*
Intention to Leave^8^	98	1.54 (0.84)	176	1.89 (0.91)	88	1.76 (0.84)	(2, 359) = 4.98	0.027	.007*
Employer Attractiveness^9^	101	22.43 (7.17)	178	22.60 (6.71)	89	23.17 (5.88)	(2, 365) = 0.33	0.002	.720
Recommendation^10^	101	7.31 (2.86)	178	6.95 (2.76)	87	7.01 (2.60)	(2, 363) = 0.56	0.003	.571

^1^[37], ^2^ [37], ^3^ [37], ^4^ [39], ^5^ [43], ^6^ [44], ^7^ [45], ^8^ [48], ^9^ [49], ^10^ [50]. Numbers in brackets refer to the corresponding sources in the reference list.

ANOVA = analysis of variance. SD = standard deviation, df = degrees of freedom, ɳ_p_^2^ = partial eta squared. *Statistically significant at p < .05.

**Table 4:** Post hoc comparisons between professional groups.

Outcomes	Professional Groups	Professional Groups	Mean Difference [95% CI]	SD	Cohen’s d	p
Cooperation between occupational groups^1^	Physicians	Nurses	0.25 [0.01; 0.48]	0.10	0.324	.034*
	Nurses	Others	−0.02 [-.0.27; 0.22]	0.10	−0.029	.994
	Others	Physicians	−0.23 [−0.50; 0.05]	0.11	−0.294	.138
Psychosocial Safety Climate^2^	Physicians	Nurses	−3.23 [−5.99; −0.47]	1.15	−0.391	.016*
	Nurses	Others	−0.25 [−3.20; 2.69]	1.23	−0.026	.996
	Others	Physicians	3.48 [0.20; 6.77]	1.37	0.395	.034*
Effort-Reward Imbalance Scale^3^	Physicians	Nurses	−0.13 [−0.23; −0.02]	0.04	−0.383	.010*
	Nurses	Others	0.11 [0.00; 0.22]	0.05	0.317	.050*
	Others	Physicians	0.02 [−0.11; 0.14]	0.05	0.050	.983
Job Satisfaction^4^	Physicians	Nurses	1.97 [0.52; 3.41]	0.60	0.404	.004*
	Nurses	Others	−0.88 [−2.38; 0.63]	0.63	−0.180	.412
	Others	Physicians	−1.09 [−2.77; 0.60]	0.70	−0.231	.324
Intention to Leave^5^	Physicians	Nurses	−0.35 [−0.61; −0.08]	0.11	−0.394	.005*
	Nurses	Others	0.13 [−0.15; 0.40]	0.11	0.146	.619
	Others	Physicians	0.22 [−0.09; 0.53]	0.13	0.261	.229

^1^[37], ^2^ [39], ^3^ [44], ^4^ [45], ^5^ [48]. Numbers in brackets refer to the corresponding sources in the reference list. CI = confidence interval, SD = standard deviation. *Statistically significant at p <.05.

### Differences between employees with and without full-time contracts (working hours)

When study participants with or without full-time contracts were analyzed, eight scales were rated significantly more positively by the full-time working employees: job decision authority, cooperation between occupational groups, the irritation scale, the psychosocial safety climate, job satisfaction, intention to leave, employer attractiveness and recommendation (Table 5). Results did not change when considering hospital as an additional factor.

**Table 5:** Working conditions, job stress and work-related consequences: comparisons between groups with or without full-time contracts. ANOVAs with effect sizes.

	Full-time working		Part-time working		Group effect		
Outcomes	n	Mean (SD)	n	Mean (SD)	F (df)	ɳ_p_^2^	p
Job decision authority^1^	240	3.36 (0.89)	131	3.03 (0.89)	(1, 369) = 11.75	0.031	<.001*
Quantitative job demands^2^	243	3.52 (0.88)	133	3.52 (0.85)	(1, 374) = 0.003	0.000	.955
Cooperation between occupational groups^3^	243	3.72 (0.79)	133	3.51 (0.77)	(1, 374) = 6.60	0.017	.011*
Psychosocial Safety Climate^4^	260	30.47 (10.49)	136	26.70 (7.48)	(1, 358) = 17.07	0.034	<.001*
Irritation Scale^5^	262	25.10 (9.06)	140	28.15 (10.13)	(1,400) = 9.52	0.023	.002*
Effort-Reward Imbalance Scale^6^	233	1.50 (0.34)	125	1.52 (0.35)	(1, 356) = 0.37	0.001	.545
Job Satisfaction^7^	243	31.03 (4.72)	133	28.99 (4.86)	(1, 374) = 15.75	0.040	<.001*
Intention to Leave^8^	239	1.70 (0.89)	131	1.90 (0.87)	(1, 368) = 4.34	0.012	.038*
Employer Attractiveness^9^	243	23.54 (6.56)	133	21.23 (6.60)	(1, 374) = 10.60	0.028	.001*
Recommendation^10^	243	7.42 (2.72)	132	6.47 (2.75)	(1, 373) = 10.49	0.027	.001*

^1^[37], ^2^ [37], ^3^ [37], ^4^ [39], ^5^ [43], ^6^ [44], ^7^ [45], ^8^ [48], ^9^ [49], ^10^ [50]. Numbers in brackets refer to the corresponding sources in the reference list.

ANOVA = analysis of variance. SD = standard deviation, df = degrees of freedom, ɳ_p_^2^ = partial eta squared. *Statistically significant at p < .05.

### Differences between employees with and without leadership positions (job level)

Employees in leadership positions rated eight of the scales significantly more positively than employees without leadership roles: job decision authority, cooperation between professional groups, psychosocial safety climate, irritation scale, job satisfaction, intention to leave, employer attractiveness and recommendation (Table 6). Results did not change when considering hospital as an additional factor.

**Table 6:** Working conditions, job stress and work-related consequences: comparisons between groups with or without leadership positions. ANOVAs with effect sizes.

	Without leadership position		With leadership position		Group effect		
Outcomes	n	Mean (SD)	n	Mean (SD)	F (df)	ɳ_p_^2^	p
Job decision authority^1^	223	3.03 (0.84)	152	3.57 (0.91)	(1, 373) = 35.43	0.040	<.001*
Quantitative job demands^2^	226	3.55 (0.90)	154	3.48 (0.81)	(1, 378) = 0.58	0.002	.448
Cooperation between occupational groups^3^	226	3.54 (0.84)	154	3.81 (0.67)	(1, 367) = 11.65	0.028	<.001*
Psychosocial Safety Climate^4^	248	27.53 (9.31)	147	31.47 (9.83)	(1, 398) = 16.51	0.040	<.001*
Irritation Scale^5^	244	26.87 (10.46)	162	25.07 (7.86)	(1, 398) = 3.93	0.009	.048*
Effort-Reward Imbalance Scale^6^	215	1.51 (0.37)	146	1.50 (0.31)	(1, 344) = 0.22	0.001	.639
Job Satisfaction^7^	226	28.83 (4.78)	154	32.41 (4.12)	(1, 378) = 57.40	0.132	<.001*
Intention to Leave^8^	222	1.87 (0.93)	152	1.62 (0.79)	(1, 372) = 7.61	0.020	.006*
Employer Attractiveness^9^	226	21.09 (6.51)	154	25.07 (6.13)	(1, 378) = 36.02	0.087	<.001*
Recommendation^10^	224	6.45 (2.69)	154	7.99 (2.60)	(1, 376) = 31.03	0.076	<.001*

^1^[37], ^2^ [37], ^3^ [37], ^4^ [39], ^5^ [43], ^6^ [44], ^7^ [45], ^8^ [48], ^9^ [49], ^10^ [50]. Numbers in brackets refer to the corresponding sources in the reference list.

ANOVA = analysis of variance. SD = standard deviation, df = degrees of freedom, ɳ_p_^2^ = partial eta squared. *Statistically significant at p < .05.

### Additional analyses: Subgroup analyses focusing on groups that deserve the most attention

Subgroup analyses are presented in Supporting File 2 (S2 File). When solely focusing on nurses (n = 192), persons with full-time contracts indicated greater job decision authority, cooperation between occupational groups, and a higher psychosocial safety climate, less irritation, more job satisfaction, and recommended their employer more than did persons with part-time contracts. Furthermore, nurses with leadership responsibilities indicated greater job decision authority, cooperation between occupational groups, and job satisfaction, rated their employer as more attractive and recommended it more than did nurses without leadership responsibilities.

When focusing on part-time workers (n = 140), other professionals indicated a greater psychosocial safety climate than did physicians and nurses. Part-time working leaders rated job decision authority, psychosocial safety, and job satisfaction as higher and rated their employer as more attractive and recommended it to a greater extent than did part-time employees without leadership responsibilities.

With respect to employees without leadership responsibilities (n = 244), nurses rated a higher psychosocial safety climate than did physicians. Nurses indicated a greater effort-reward imbalance than other professionals did. Furthermore, part-time employees without leadership responsibility rated the psychosocial safety climate lower than full-time employees.

## Discussion

This research examined staff perceptions at three German hospitals, utilizing a comprehensive survey approach to assess working conditions, job stress, and work-related consequences across multiple healthcare professionals. Compared to reference data [5,43,53–56], our hospital sample data showed suboptimal outcomes, particularly in quantitative job demands, psychosocial safety climate, irritation, effort-reward imbalance, and intention to leave. The analysis revealed significant disparities in perceived working conditions and stress levels among different professional groups. Nursing staff emerged as the group with the highest need for improvement in working conditions, reporting higher levels of work-related stress and less favorable perceptions of their working environment. Compared with their full-time counterparts, part-time employees expressed greater need for improvements in working conditions. Nonmanagerial staff reported more negative perceptions of working conditions and higher stress levels than did those in leadership positions. By identifying groups that deserve the most attention and key areas of improvement, this study provides valuable insights for hospital administrators seeking to create more supportive and attractive work environments.

Unlike other studies that examine professional groups [5,16,17], no significant differences were observed regarding the working conditions of job decision authority and quantitative work demands. This could be due to the dismantling of hierarchies in the hospital sector or due to differences regarding the measurement instrument. The generally high level of temporal overload across all occupational groups was in line with other studies [4,5,57].

In contrast to previous findings [5,18], physicians reported more positive assessments of cooperation than nurses did. This may be attributed to their social standing and appreciation, and medical curricula may have increased emphasis on teamwork [58], fostering a more positive view of cooperation. Nursing curricula typically emphasize intra-team collaboration more than explicit interdisciplinary physician-nurse training [59]. In contrast, nurses and other occupations experienced a more positive psychosocial safety climate than did physicians, despite the overall evaluation indicating a high level of risk concerning occupational health and safety [40]. The psychosocial safety climate is particularly salient, as it precedes and influences working conditions [60]. The paradox—physicians’ better cooperation but poorer PSC—likely reflects role-specific contexts and demands. It may arise from physicians’ high-stakes autonomy and hierarchical pressures, whereas nurses might perceive stronger organizational team support but rate interpersonal dynamics lower potentially due to frontline challenges amid staffing shortages [61,62]. These cross-sectional findings cannot establish causality between PSC and cooperation; longitudinal data could clarify temporal dynamics. Furthermore, factors related to PSC, such as corporate communication about mental health or the participation of employees in stress prevention, were found to be negatively correlated with depressive symptoms [63]. To date, perceptions of the psychosocial safety climate in the hospital sector have remained largely unexamined. These results underscore a need for improvement in risk management related to the work environment, especially for physicians. As a consequence, the mental health of hospital employees should be a higher priority for hospital management.

Consistent with other studies regarding satisfaction with remuneration [17,22], nurses reported the highest effort–reward imbalance. In line with previous research [5,16,17], physicians demonstrated greater job satisfaction and lower intentions to leave than nurses did. For nurses, factors such as organizational commitment, social community, work-life conflicts, leadership qualities, opportunities for professional development, role conflicts, and inadequate rewards contribute to job dissatisfaction [17].

These results collectively suggest that physicians evaluate working conditions, job stress and work-related consequences at the individual and organizational levels more positively than do nurses. One possible explanation might be that physicians are less dependent upon leadership behavior than other professional groups are [7,64]. Within the nursing group, those with full-time contracts and leadership responsibilities reported fewer work-related challenges. These results align with those of the RN4CAST study [65], where across nine out of twelve European countries, including Germany, more than half of the surveyed nurses assessed their working environment as poor or fair. An increase in nurses’ workload by only one patient increased the probability of an inpatient dying within 30 days of admission. Therefore, nursing services in particular should be supported to ensure high-quality health care [66]. Given that, in 2022, nursing staff constituted the second largest workforce (39%) in German hospitals, surpassed only by other professions (43%) and significantly outnumbered medical doctors (18%) [67], these results warrant serious consideration from both hospital management and policymakers. To counteract the ongoing threatening intention to leave, which was also confirmed within this study, implications need to be urgently drawn.

Unlike previous studies that examined working hours in medical practices [23,24], the present study was focused on a hospital setting and identified significant disparities. Full-time workers rated the working conditions of job decision authority, cooperation between occupational groups, and psychosocial safety climate, the irritation scale, and the work-related consequences of job satisfaction, intention to leave, employer attractiveness and recommendation as more positive. Among part-time working employees, leadership is a protective factor, and other professionals rated the psychosocial safety climate as more positive than did nurses or physicians. These results are in line with previous research showing that part-time work might be associated with more work density, not reducing the risk for burnout [68]. This is in contrast to a Swiss study showing that working full-time was associated with a higher risk of poor well-being [26]. Another study investigating burnout and work-family conflict among physicians working in hospital and ambulatory care settings found that part-time employees reported more favorable work-family conflict outcomes but no differences in level of burnout [25]. In this survey conducted in Saxony, Germany, 31.8% of physicians worked part-time. Considering that 42% of hospital staff in Germany are employed part-time [69], these results have important implications for hospital management. Moreover, there appears to be a growing tendency among physicians to work part-time. For example, more than 80% of physicians beginning their medical training at a university hospital in the Netherlands expressed a desire to reduce their working hours [70]. However, this significant segment of the workforce is often neglected. The results suggest that enhancing the involvement of part-time employees may be crucial regarding the on-going trend.

This study included both leaders and non-leaders from all occupational groups within hospitals. This broader inclusion revealed significant differences. As expected [27,30], employees in leadership positions reported significantly more favorable outcomes regarding job decision authority, interprofessional cooperation, psychosocial safety climate, irritation, job satisfaction, intention to leave, employer attractiveness, and willingness to recommend their employer. Interestingly, the findings diverged from those of a systematic review [71], which posited that physician leaders experience increased overtime due to managerial responsibilities supplementing their clinical duties. In contrast to expectations [31], the current study did not identify significant disparities in temporal overload between leaders and non-leaders, indicating however a high level of quantitative work demands across all groups. In line with other studies [7,31], followers tended to have lower levels of well-being than leaders had. Among employees without leadership roles, nurses and part-time professionals—who form the largest part of the workforce—require special attention. Nurses reported a less favorable effort-reward balance. Physicians and part-time workers reported a less favorable psychosocial safety climate.

The study evaluated for the first time several workplace factors for various hospital employees in Germany. Identifying the most significant workplace stressors is essential for developing effective prevention strategies [72,73]. Focus group discussions with hospital staff in England showed that listening to employees, building trust, and understanding their needs are crucial first steps [74]. While there is growing evidence supporting workplace mental health interventions [75], most focus on individual workers and illnesses rather than on improving working conditions. Our research responds to this imbalance by emphasizing the need for tailored, structural interventions addressing the specific challenges faced by different hospital employee groups. Overall, it seems necessary to support the nursing service as well as part-time working employees – especially in a non-leadership position – without neglecting the other groups in the hospital setting. To address these disparities effectively, the following approaches are recommended: implementing holistic interventions that combine both behavioral and relational-preventive elements [76], such as the “SEEGEN” program [35], considering the impact of public health [77], developing targeted support mechanisms for nursing staff, addressing their specific challenges related to effort‒reward imbalance and job satisfaction, designing flexible work arrangements and support systems for part-time employees, and taking mental health more into consideration to improve the overall psychosocial safety climate and strengthen leadership development to expand its influence on all employees.

Occupational science research on work and health has repeatedly shown that social support and good social relationships are among the central health-promoting resources for employees [64,78,79]. Healthcare organizations must acknowledge their critical role in cultivating a supportive work culture and environment to enhance employee well-being [80–83].

Several limitations apply to this study. The results are based on a cross-sectional approach. There might be a selection bias regarding those participants most interested in mental health. The results are limited with respect to generalizability. For privacy reasons, no hospital-specific evaluations could be carried out. Data were analyzed at the individual level; low intraclass correlations (<0.05) suggest that hospital-level clustering effects were minimal. Future studies may benefit from multilevel modelling when hospital-level variance is substantial, and samples are more balanced across sites. Additionally, the study represents an exploratory approach with multiple tests. For a combination of factors such as professional groups, working hours and job level, a larger sample size is needed in future studies [52]. Moreover, the subscales of the TAA-KH-S were shortened for practical reasons. Therefore, the results need to be interpreted with caution. Potential confounding factors, such as sex, were deliberately not controlled for, as they are considered inherent characteristics of the target population in nonrandomized trials [52]. In addition, a differentiation between more than two hierarchical levels was not undertaken [31]. Differences may exist between part-time employees working 15–34 hours versus less than 15 hours per week. Future studies should further differentiate working hours.

## Conclusions

The study combined in a naturalistic setting, the assessment of the topics of working conditions, job stress, and work-related consequences from the perspective of different professional groups, working hours and job levels within hospital settings.

This provides an integrative overview of where improvements may be needed in terms of temporal overload, the psychosocial safety climate, irritation, effort-reward imbalance and the intention to leave across all groups. There were, in part, large differences in the evaluation of those topics. Nurses emerged as the professional group with the greatest need for improvement in working conditions, exhibiting higher levels of effort-reward imbalance, lower job satisfaction and elevated intentions to leave their positions. Furthermore, one often neglected workforce group – part-time working employees – reported lower job decision authority, reduced cooperation between occupational groups, diminished perceptions of a psychosocial safety climate, higher levels of irritation and dissatisfaction, increased intention to leave, and lower perceived employer attractiveness and recommendation rates. Employees in leadership positions generally reported more favorable occupational health outcomes, suggesting that leadership roles may serve as a protective factor against work-related stress and dissatisfaction.

While this research provided an overview focusing on single effects that are easily understandable, future studies should shed more light on the combination of those relevant group factors.

The findings highlight the importance of considering professional groups, working hours, and job levels when designing occupational health measures in hospital settings. A nuanced, multifaceted approach is necessary to address the diverse needs of different employee groups, with particular attention given to nursing staff and part-time workers.

Each healthcare professional has an impact on the quality of care and patient safety and health [66]. Therefore, the most important way to support the healthcare system is by assisting each professional in doing his or her best. This approach has the potential to enhance both employee satisfaction and patient outcomes, ultimately contributing to a more robust and effective healthcare system.

## Supporting information

S1 File Questionnaire and survey guide. It contains the instruments used for analysis in this study and additional information.
(PDF)

S2 File Additional analyses: Subgroup analyses. Subgroup analyses focus on the groups that deserve the most attention: nurses, part-time working employees and employees without leadership responsibilities.
(PDF)
