# Supplementary material for: Working conditions, job stress and work-related consequences among hospital employees—differences by professional group, working hours and job levels: A cross-sectional study
Source: PLoS One. 2026 Mar 12;21(3):e0343567. doi: 10.1371/journal.pone.0343567 (PMC12981435; doi:10.1371/journal.pone.0343567)
Supplement: S1 File — It contains the instruments used for analysis in this study and additional information. (PDF) [file pone.0343567.s001.pdf]

S1 File. Questionnaire and Survey Guide.

It contains the instruments used for analysis in this study and additional information.

General Information
This document contains the instruments used for the analysis in this study.
All instruments are provided in English. For some scales, no official English translation was available; in these cases, the translation was carried out by the study authors and is clearly indicated below. References to the original (German) versions are provided. Any shortened or modified versions are clearly noted. The order of the items corresponds to the sequence in which they were presented to participants in the questionnaire. The items as shown in the boxes below are as they were presented to participants. Details on data analysis are provided in the Methods section of the manuscript.

Table of Contents

- Sociodemographic Questions
- [Scale: IRR – Irritation Scale]
- [Scale: PSC-12 – Psychosocial Safety Climate
- [Scale: Job Satisfaction – the General Job Satisfaction Scale
- [Scale: Intention to leave]
- [Scale: ERI – Effort-Reward Imbalance Questionnaire]
- [Scale: TAA-KH-S – Screening Work Analysis Instrument for Hospitals-Self Report Version]
- [Scale: Recommendation]
- [Scale: Employer Attractiveness]

date |_|_|.|_|_|.|_|_|_|_|

Questions about you:

1 You are:

1 ☐ Male
2 ☐ Female
3 ☐ Diverse

2 Age _________ years

3 Marital status (Please select only one):

1 ☐ married / in a committed partnership
3 ☐ divorced
2 ☐ single
4 ☐ widowed

4 Which best describes your current employment status? (Please select only one):

1 ☐ Employed full-time
3 ☐ Maternity, parental, educational or other leave
2 ☐ Employed part-time, percentage: _________%
4 ☐ Other, please specify: ______________________________

Questions about your professional activities:

5 Which occupational group do you belong to? (Please select only one):

1 ☐ Medical staff
2 ☐ Medical-technical staff
3 ☐ Nursing staff
4 ☐ Functional service
5 ☐ Secretarial staff
6 ☐ Other, please specify: ______________________________

6 Do you work in shifts?

1 ☐ Yes
2 ☐ No

7 Do you have personnel responsibility?

1 ☐ Yes
2 ☐ No

7b If yes, for how many people in total (direct and indirect): |__|__|__|__|

8 Which of the following three hierarchical levels best describes your current position?

1 ☐ Overall management responsibility for an entire department (e.g., chief physician, administrative manager, board member of the entire hospital)
3 ☐ Employee with management responsibility for subareas (e.g., senior physician, head of nursing, team leader)
2 ☐ Employee without management responsibilty

9 How long have you been working professionally? For |__|__| years or since the year |__|__|__|__|

Translation note: Translated by the study authors; no official English version available.

Please answer the questions openly and honestly, and without consulting your colleagues. Go through the questions in order and mark the answer box that best matches your opinion in each case.

IRR – Irritation Scale

Please read each statement carefully and indicate how often you have felt this way in the past few months. Mark the box that best describes your situation.

		Strongly disagree<br>1	Disagree<br>2	Somewhat disagree<br>3	Neither agree or disagree<br>4	Somewhat agree<br>5	Agree<br>6	Strongly agree<br>7
1	I have difficulty relaxing after work.	☐	☐	☐	☐	☐	☐	☐
2	Even at home I often think of my problems at work.	☐	☐	☐	☐	☐	☐	☐
3	I get grumpy when others approach me.	☐	☐	☐	☐	☐	☐	☐
4	Even on my vacations I think about my problems at work.	☐	☐	☐	☐	☐	☐	☐
5	From time to time I feel like a bundle of nerves.	☐	☐	☐	☐	☐	☐	☐
6	I anger quickly.	☐	☐	☐	☐	☐	☐	☐
7	I get irritated easily, although I don’t want this to happen.	☐	☐	☐	☐	☐	☐	☐
8	When I come home tired after work, I feel rather irritable.	☐	☐	☐	☐	☐	☐	☐

Original reference: Mohr G, Rigotti T, Müller A. Irritation - ein Instrument zur Erfassung psychischer Beanspruchung im Arbeitskontext. Skalen- und Itemparameter aus 15 Studien [Irritation - an instrument assessing mental strain in working contexts. Scale and item parameters from 15 studies]. Zeitschrift für Arbeits- und Organ A&O. 2005;49(1):44–8.

English adaptation: Mohr G, Müller A, Rigotti T, Aycan Z, Tschan F. The assessment of psychological strain in work contexts. European Journal of Psychological Assessment. 2006; 22(3): 198-206.

PSC-12 – Psychosocial Safety Climate

Please indicate your level of agreement with each statement regarding your workplace. Mark the box that best matches your experience.

		Strongly disagree<br>1	Disagree<br>2	Neither agree or disagree<br>3	Agree<br>4	Strongly agree<br>5
1	In my workplace senior management acts quickly to correct problems/issues that affect employees' psychological health.	☐	☐	☐	☐	☐
2	Senior management acts decisively when a concern of an employees' psychological status is raised.	☐	☐	☐	☐	☐
3	Senior management show support for stress prevention through involvement and commitment.	☐	☐	☐	☐	☐
4	Psychological well-being of staff is a priority for this organization	☐	☐	☐	☐	☐
5	Senior management clearly considers the psychological health of employees to be of great importance.	☐	☐	☐	☐	☐
6	Senior management considers employee psychological health to be as important as productivity.	☐	☐	☐	☐	☐
7	There is good communication here about psychological safety issues which effect me.	☐	☐	☐	☐	☐
8	Information about workplace psychological well-being is always brought to my attention by my manager/supervisor.	☐	☐	☐	☐	☐
9	My contributions to resolving occupational health and safety concerns in the organization are listened to.	☐	☐	☐	☐	☐
10	Participation and consultation in psychological health and safety occurs with employees', unions and health and safety representatives in my workplace.	☐	☐	☐	☐	☐
11	Employees are encouraged to become involved in psychological safety and health matters.	☐	☐	☐	☐	☐
12	In my organization, the prevention of stress involves all levels of the organization.	☐	☐	☐	☐	☐

Original reference: Hall GB, Dollard MF, Coward J. Psychosocial safety climate: Development of the PSC-12. Int J Stress Manag. 2010;17(4):353–83.

Job Satisfaction – the General Job Satisfaction Scale

Please indicate to what extent you agree with the following statements.

		false<br>1	Mostly false<br>2	Neither true nor false<br>3	Mostly true<br>4	true<br>5
1	My work gives me little pleasure, but one shouldn't expect too much.	☐	☐	☐	☐	☐
2	I really enjoy my work.	☐	☐	☐	☐	☐
3	My work is always the same routine; there's nothing one can do about it.	☐	☐	☐	☐	☐
		Very uninteresting<br>1	Quite uninteresting<br>2	Neither interesting nor uninteresting<br>3	Quite interesting<br>4	Yes, very interesting<br>5
4	What do you think: All things considered, would you say that your work is really interesting and satisfying?	☐	☐	☐	☐	☐
		Very few opportunities<br>1	Quite few opportunities<br>2	Neither nor<br>3	Quite a lot of opportunities<br>4	Yes, very many opportunities<br>5
5	Does your work give you enough opportunities to use your abilities?	☐	☐	☐	☐	☐
		Very dissatisfied<br>1	Quite dissatisfied<br>2	Neither satisfied nor dissatisfied<br>3	Quite satisfied<br>4	Yes, very satisfied<br>5
6	Are you satisfied with your opportunities for advancement?	☐	☐	☐	☐	☐
7	Are you satisfied with the pace of work?	☐	☐	☐	☐	☐
		Definitely not<br>1	Probably not<br>2	Neither nor<br>3	Probably<br>4	Yes, definitely<br>5
8	If you had to decide again, would you choose the same profession?	☐	☐	☐	☐	☐

Original reference: Fischer L, Lück HE. Allgemeine Arbeitszufriedenheit: Zusammenstellung sozialwissenschaftlicher Items und Skalen [General Job Satisfaction: Compilation of Social Science Items and Scales]. 2001. https://zis.gesis.org/skala/Fischer-Lück-Allgemeine-Arbeitszufriedenheit. Accessed 16 Feb 2025.

Short version: Fischer L, Eufinger A. Zur Differenzierung von Formen der Arbeitszufriedenheit mit unterschiedlichen Messverfahren. In: Fischer L, Borg I, editors. Arbeitszufriedenheit: Beiträge zur Organisationspsychologie. 5th ed. Stuttgart: Verlag für Angewandte Psychologie; 1991. p. 115–32.

Translation note: Translated by the study authors; no official English version available.

Intention to Leave

Please indicate the answer that applies to you.

		Never<br>1	A few times a year<br>2	A few times a month<br>3	A few times a week<br>4	Every day<br>5
1	How often during the previous twelve months have you thought of leaving your profession?	☐	☐	☐	☐	☐

Original references: Simon M, Tackenberg P, Hasselhorn HM, Kümmerling A, Büscher A, Müller BH. Auswertung der ersten Befragung der NEXT-Studie in Deutschland [Analysis of the first survey of the NEXT study in Germany]. 2005. http://www.next.uni-wuppertal.de/. Accessed 13 Feb 2025.
Hämmig O. Explaining burnout and the intention to leave the profession among health professionals – a cross-sectional study in a hospital setting in Switzerland. BMC Health Serv Res. 2018;18:785. https://doi.org/10.1186/s12913-018-3556-1.

Translation note: Authors' translation adapted from Hämmig (2018)

ERI – Effort-Reward Imbalance Questionnaire

Please indicate to what extent you agree with the following statements:

		Strongly disagree 1	Disagree 2	Agree 3	Strongly agree 4
1	I have constant time pressure due to a heavy workload.	☐	☐	☐	☐
2	I have many interruptions and disturbances while performing my job.	☐	☐	☐	☐
3	Over the past few years, my job has become more and more demanding.	☐	☐	☐	☐
4	I receive the respect I deserve from my superior or a respective relevant person.	☐	☐	☐	☐
5	My job promotion prospects are poor.	☐	☐	☐	☐
6	I have experienced or I expect to experience an undesirable change in my work situation.	☐	☐	☐	☐
7	My job security is poor.	☐	☐	☐	☐
8	Considering all my efforts and achievements, I receive the respect and prestige I deserve in my work.	☐	☐	☐	☐
9	Considering all my efforts and achievements, my promotion prospects are adequate.	☐	☐	☐	☐
10	Considering all my efforts and achievements, my salary/income is adequate.	☐	☐	☐	☐

Original reference: Siegrist J, Wege N, Pühlhofer F, Wahrendorf M. A short generic measure of work stress in the era of globalization: Effort-reward imbalance. Int Arch Occup Environ Health. 2009;82(8):1005–13.

TAA-KH-S – Screening Work Analysis Instrument for Hospitals-Self Report Version

Please indicate how often each of the following statements applies to your work situation by marking the appropriate answer.

**Job decision authority**	No, not at all<br>1	Rather no<br>2	Partly<br>3	Rather yes<br>4	Yes, to a very great extent 5
1 I can decide for myself how to carry out my work.	☐	☐	☐	☐	☐
2 I have opportunities to make my own decisions when determining tasks.	☐	☐	☐	☐	☐
3 I can shape my tasks according to my own ideas.	☐	☐	☐	☐	☐
**Quantitative job demands**	No, not at all<br>1	Rather no<br>2	Partly<br>3	Rather yes<br>4	Yes, absolut ely<br>5
4 I repeatedly have to rush, yet I still do not manage to finish my work.	☐	☐	☐	☐	☐
5 I often have too much to do at once in my work.	☐	☐	☐	☐	☐
6 Due to short-term deadlines, I repeatedly experience time pressure at work.	☐	☐	☐	☐	☐
**Cooperation between occupational groups**	No, not at all<br>1	Rather no<br>2	Partly<br>3	Rather yes<br>4	Yes, absolut ely<br>5
7 There is a relationship of trust among colleagues in my department.	☐	☐	☐	☐	☐
8 There is a relationship of trust with employees from other departments/services in the organization.	☐	☐	☐	☐	☐

Original references: Büssing A, Glaser J, Höge T. Screening psychischer Belastungen in der stationären Krankenpflege (Belastungsscreening TAA-KH-S). Manual und Materialien [Screening of Psychological Stress in Inpatient Nursing (Stress Screening TAA-KH-S). Manual and Materials]. Diagnostica. 2001;47(2):77–87.
Elke G. Rezension des Tätigkeits- und Arbeitsanalyseverfahrens für das Krankenhaus - Selbstbeobachtungsversion (TAA-KH-S) von André Büssing und Jürgen Glaser [Screening Work Analysis Instrument for Hospitals-Self Report (TAA-KH-S) by André Büssing and Jürgen Glaser]. Zeitschrift für Arbeits- und Organ A&O. 2004;48(3):148–53.

Adapted version: (1) Job decision authority (shortened from nine to three items), (2) Quantitative job demands (three items) and (3) Cooperation between occupational groups (shortened from five to two items).

Translation note: Translated by the study authors; no official English version available.

Recommendation

The following question refers to the recommendation of your company. Please indicate to what extent you agree with the following statement.

		Highly unlikely										Highly likely
		1	2	3	4	5	6	7	8	9	10	11
1	How likely is it that you would recommend this company to a friend or colleague?	☐	☐	☐	☐	☐	☐	☐	☐	☐	☐	☐

Original reference: Reichheld FE. The one number you need to grow. Harv Bus Rev. 2003;81:46–54.
Krol MW, de Boer D, Delnoij DM, Rademakers JJDJM. The Net Promoter Score - an asset to patient experience surveys? Heal Expect. 2015;18(6):3099–109.

Adapted version: Modified version (“this” instead of “our”)

Employer Attractiveness

Please indicate to what extent you agree with the following statements about your employer (your hospital).

		Strongly disagree						Strongly agree
		1	2	3	4	5	6	7
1	I enjoy working for my employer.	☐	☐	☐	☐	☐	☐	☐
2	I have a positive attitude towards my employer.	☐	☐	☐	☐	☐	☐	☐
3	I prefer my employer over other comparable employers (competitors).	☐	☐	☐	☐	☐	☐	☐
4	I feel emotionally attached to my employer.	☐	☐	☐	☐	☐	☐	☐
5	I find my employer attractive.	☐	☐	☐	☐	☐	☐	☐

Original reference: Bruhn M, Batt V, Flückiger B. Aufbau von Arbeitgeberattraktivität - Identifikation der Determinanten und empirische Überprüfung. [Building employer attractiveness - identification of determinants and empirical verification]. Die Unternehmung. 2013;67(1):62–82.

Translation note: Translated by the study authors; no official English version available.

References

Bruhn M, Batt V, Flückiger B. Aufbau von Arbeitgeberattraktivität - Identifikation der Determinanten und empirische Überprüfung. [Building employer attractiveness - identification of determinants and empirical verification]. Die Unternehmung. 2013;67(1):62–82.

Büssing A, Glaser J, Höge T. Screening psychischer Belastungen in der stationären Krankenpflege (Belastungsscreening TAA-KH-S). Manual und Materialien [Screening of Psychological Stress in Inpatient Nursing (Stress Screening TAA-KH-S). Manual and Materials]. Diagnostica. 2001;47(2):77–87.

Elke G. Rezension des Tätigkeits- und Arbeitsanalyseverfahrens für das Krankenhaus - Selbstbeobachtungsversion (TAA-KH-S) von André Büssing und Jürgen Glaser [Screening Work Analysis Instrument for Hospitals-Self Report (TAA-KH-S) by André Büssing and Jürgen Glaser]. Zeitschrift für Arbeits- und Organ A&O. 2004;48(3):148–53.

Fischer L, Eufinger A. Zur Differenzierung von Formen der Arbeitszufriedenheit mit unterschiedlichen Messverfahren. In: Fischer L, Borg I, editors. Arbeitszufriedenheit: Beiträge zur Organisationspsychologie. 5th ed. Stuttgart: Verlag für Angewandte Psychologie; 1991. p. 115–32.

Fischer L, Lück HE. Allgemeine Arbeitszufriedenheit: Zusammenstellung sozialwissenschaftlicher Items und Skalen [General Job Satisfaction: Compilation of Social Science Items and Scales] [Internet], 2001 [cited 2025 JUN 17]. Available from: https://doi.org/10.6102/zis1

Hall GB, Dollard MF, Coward J. Psychosocial safety climate: Development of the PSC-12. Int J Stress Manag. 2010;17(4):353–83.

Hämmig O. Explaining burnout and the intention to leave the profession among health professionals – a cross-sectional study in a hospital setting in Switzerland. BMC Health Serv Res. 2018;18:785. https://doi.org/10.1186/s12913-018-3556-1

Krol MW, de Boer D, Delnoij DM, Rademakers JJDJM. The Net Promoter Score - an asset to patient experience surveys? Heal Expect. 2015;18(6):3099–109. doi: 10.1111/hex.12297.

Mohr G, Müller A, Rigotti T, Aycan Z, Tschan F. The assessment of psychological strain in work contexts. European Journal of Psychological Assessment. 2006; 22(3): 198-206.

Mohr G, Rigotti T, Müller A. Irritation - ein Instrument zur Erfassung psychischer Beanspruchung im Arbeitskontext. Skalen- und Itemparameter aus 15 Studien [Irritation - an instrument assessing mental strain in working contexts. Scale and item parameters from 15 studies]. Zeitschrift für Arbeits- und Organ A&O. 2005;49(1):44–8.

Reichheld FE. The one number you need to grow. Harv Bus Rev. 2003;81:46–54.

Siegrist J, Wege N, Pühlhofer F, Wahrendorf M. A short generic measure of work stress in the era of globalization: Effort-reward imbalance. Int Arch Occup Environ Health. 2009;82(8):1005–13.

Simon M, Tackenberg P, Hasselhorn HM, Kümmerling A, Büscher A, Müller BH. Auswertung der ersten Befragung der NEXT-Studie in Deutschland [Evaluation of the first survey of the NEXT study in Germany] [Internet]; 2005 [cited 2025 JUL 21]. Available from: http://www.next.uni-wuppertal.de/
