# Supplementary material for: Working conditions, job stress and work-related consequences among hospital employees—differences by professional group, working hours and job levels: A cross-sectional study
Source: PLoS One. 2026 Mar 12;21(3):e0343567. doi: 10.1371/journal.pone.0343567 (PMC12981435; doi:10.1371/journal.pone.0343567)
Supplement: S2 File — Subgroup analyses focus on the groups that deserve the most attention: nurses, part-time working employees and employees without leadership responsibilities. (PDF) [file pone.0343567.s002.pdf]

S2 File. Additional analyses: subgroup analyses.

Subgroup analyses focus on the groups that deserve the most attention: nurses, part-time working employees and employees without leadership.

a) Nurses

Tabe 1s: Working conditions, job stress and work-related consequences: comparisons between groups with or without full-time contracts among nurses. ANOVAs with effect sizes.

	Full-time working		Part-time working		Group effect		
**Outcomes**	n	Mean (SD)	n	Mean (SD)	F (df)	η_p^2	p
Job decision authority[1]	107	3.29 (0.83)	70	2.95 (0.80)	(1, 175) = 7.33	0.040	.007*
Quantitative job demands[2]	107	3.60 (0.89)	71	3.50 (0.86)	(1, 176) = 0.52	0.003	.470
Cooperation between occupational groups[3]	107	3.68 (0.83)	71	3.41 (0.74)	(1, 176) = 5.08	0.028	.025*
Psychosocial Safety Climate[4]	116	32.79 (11.40)	75	25.64 (6.30)	(1, 185.11) = 30.97	0.115	< .001*
Irritation Scale[5]	116	23.74 (9.52)	76	28.13 (9.91)	(1,190) = 9.45	0.047	.002*
Effort-Reward Imbalance Scale[6]	102	1.60 (0.34)	69	1.60 (0.33)	(1, 169) = 0.00	0.000	.992
Job Satisfaction[7]	107	30.35 (5.00)	71	28.34 (4.68)	(1, 176) = 7.25	0.040	.008*
Intention to Leave[8]	106	1.84 (0.95)	70	1.96 (0.86)	(1, 174) = 0.698	0.004	.405
Employer Attractiveness[9]	107	23.38 (6.66)	71	21.43 (6.67)	(1, 176) = 3.64	0.020	.058
Recommendation[10]	107	7.45 (2.68)	71	6.20 (2.77)	(1, 176) = 9.08	0.049	.003*

[1] [37], [2] [37], [3] [37], [4] [39], [5] [43], [6] [44], [7] [45], [8] [48], [9] [49], [10] [50]. Numbers in brackets refer to the corresponding sources in the reference list.

ANOVA = analysis of variance. SD = standard deviation, df = degrees of freedom, η_p^2 = partial eta squared. *Statistically significant at $p < .05$.

Table 2s. Working conditions, job stress and work-related consequences: comparisons between groups with or without leadership positions among nurses. ANOVAs with effect sizes.

	Without leadership position		With leadership position		Group effect		
**Outcomes**	n	Mean (SD)	n	Mean (SD)	F (df)	η_p^2	p
Job decision authority[1]	223	3.03 (0.84)	152	3.57 (0.91)	(1, 175) = 9.27	0.050	.003*
Quantitative job demands[2]	226	3.55 (0.90)	154	3.48 (0.81)	(1, 130.19) = 0.035	0.000	.852
Cooperation between occupational groups[3]	226	3.54 (0.84)	154	3.81 (0.67)	(1, 176) = 11.77	0.063	< .001*
Psychosocial Safety Climate[4]	135	28.47 (9.69)	56	33.62 (10.92)	(1, 189) = 10.37	0.052	.002*
Irritation Scale[5]	136	26.04 (10.45)	56	24.11 (8.30)	(1, 190) = 1.52	0.008	.218

Effort-Reward Imbalance Scale[6]	215	1.51 (0.37)	146	1.50 (0.31)	(1, 169) = 0.430	0.003	.513
Job Satisfaction[7]	123	28.37 (4.83)	55	32.16 (4.23)	(1, 176) = 25.18	0.125	< .001*
Intention to Leave[8]	121	1.95 (0.95)	55	1.75 (0.82)	(1, 174) = 1.92	0.011	.168
Employer Attractiveness[9]	123	21.01 (6.48)	55	26.16 (5.85)	(1, 176) = 25.54	0.127	< .001*
Recommendation[10]	123	6.31 (2.67)	55	8.38 (2.48)	(1, 176) = 23.97	0.120	< .001*

[1] [37], [2] [37], [3] [37], [4] [39], [5] [43], [6] [44], [7] [45], [8] [48], [9] [49], [10] [50]. Numbers in brackets refer to the corresponding sources in the reference list.

ANOVA = analysis of variance. SD = standard deviation, df = degrees of freedom, η_p^2 = partial eta squared. *Statistically significant at $p < .05$.

b) Part-time working employees

Table 3s. Working conditions, job stress and work-related consequences: comparisons between professional groups among part-time workers. ANOVAs with effect sizes.

	Physicians		Nurses		Others		Group effect		
**Outcomes**	n	Mean (SD)	n	Mean (SD)	n	Mean (SD)	F (df)	η_p^2	p
Job decision authority[1]	22	3.31 (1.02)	70	2.95 (0.80)	37	2.99 (0.95)	(2, 126) = 1.47	.023	.234
Quantitative job demands[2]	22	3.64 (0.98)	71	3.50 (0.86)	38	3.46 (0.72)	(2, 128) = 0.380	.006	.684
Cooperation between occupational groups[3]	22	3.75 (0.83)	71	3.41 (0.74)	38	3.55 (0.80)	(2, 128) = 1.73	.026	.181
Psychosocial Safety Climate[4]	22	24.62 (8.36)	75	25.64 (6.30)	37	30.39 (8.14)	(2, 131) = 6.58	.091	.002*
Irritation Scale[5]	22	28.00 (11.85)	75	28.13 (9.92)	37	28.23 (9.45)	(2, 135) = 0.00	.000	.996
Effort-Reward Imbalance Scale[6]	21	1.38 (0.26)	69	1.57 (0.33)	33	1.49 (0.40)	(2, 120) = 2.62	.042	.077
Job Satisfaction[7]	22	30.48 (6.00)	71	28.34 (4.68)	38	29.34 (4.37)	(2, 128) = 1.79	.027	.171
Intention to Leave[8]	21	1.79 (1.08)	70	1.96 (0.86)	38	1.82 (0.77)	(2, 126) = 0.47	.007	.625
Employer Attractiveness[9]	22	19.73 (8.07)	71	21.43 (6.67)	38	22.09 (5.24)	(2, 128) = 0.92	.014	.401
Recommendation[10]	22	7.05 (3.18)	71	6.20 (2.77)	37	6.78 (2.37)	(2, 127) = 1.07	.017	.346

[1] [37], [2] [37], [3] [37], [4] [39], [5] [43], [6] [44], [7] [45], [8] [48], [9] [49], [10] [50]. Numbers in brackets refer to the corresponding sources in the reference list.

ANOVA = analysis of variance. SD = standard deviation, df = degrees of freedom, η_p^2 = partial eta squared. *Statistically significant at $p < .05$.

Table 4s. Hochberg's GT2 post hoc comparisons between professional groups among part-time workers.

Outcomes	Professional Groups	Professional Groups	Mean Difference [95% CI]	SD	Cohen's d	p
Psychosocial Safety Climate[1]	Physicians	Nurses	-1.02 [-5.24; 3.20]	1.75	-0.150	.914
	Nurses	Others	-4.75 [-8.25; -1.25]	1.45	-0.683	.004*
	Others	Physicians	5.77 [1.08; 10.46]	1.94	0.702	.010*

[1] [39]. Numbers in brackets refer to the corresponding sources in the reference list.CI = confidence interval, SD = standard deviation. *Statistically significant at p < .05.

Table 5s. Working conditions, job stress and work-related consequences: comparisons between groups with or without leadership positions among part-time workers. ANOVAs with effect sizes.

	Without leadership position		With leadership position		Group effect		
**Outcomes**	n	Mean (SD)	n	Mean (SD)	F (df)	η_p^2	p
Job decision authority[1]	103	2.95 (0.85)	28	3.35 (0.95)	(1, 129) = 4.83	0.036	.030*
Quantitative job demands[2]	105	3.52 (0.86)	28	3.54 (0.82)	(1, 131) = 0.015	0.000	.903
Cooperation between occupational groups[3]	105	3.44 (0.78)	28	3.75 (0.73)	(1, 131) = 3.55	0.026	.062
Psychosocial Safety Climate[4]	107	25.90 (7.29)	29	29.66 (7.55)	(1, 134) = 5.97	0.043	.016*
Irritation Scale[5]	111	28.27 (10.40)	29	27.69 (9.17)	(1, 138) = 0.075	0.001	.784
Effort-Reward Imbalance Scale[6]	98	1.52 (0.37)	27	1.50 (0.27)	(1, 123) = 0.104	0.001	.748
Job Satisfaction[7]	105	28.47 (4.78)	28	30.94 (4.73)	(1, 131) = 5.94	0.043	.016*
Intention to Leave[8]	103	1.92 (0.88)	28	1.82 (0.82)	(1, 129) = 0.278	0.002	.599
Employer Attractiveness[9]	105	20.57 (6.51)	28	23.71 (6.46)	(1, 131) = 5.17	0.038	.025*
Recommendation[10]	104	6.18 (2.71)	28	7.54 (2.67)	(1, 130) = 5.50	0.041	.020*

[1] [37], [2] [37], [3] [37], [4] [39], [5] [43], [6] [44], [7] [45], [8] [48], [9] [49], [10] [50]. Numbers in brackets refer to the corresponding sources in the reference list.

ANOVA = analysis of variance. SD = standard deviation, df = degrees of freedom, η_p^2 = partial eta squared. *Statistically significant at p < .05.

c) Employees without leadership responsibility

Table 6s. Working conditions, job stress and work-related consequences: comparisons between professional groups among employees without leadership responsibility. ANOVAs with effect sizes.

	Physicians		Nurses		Others		Group effect		
**Outcomes**	n	Mean (SD)	n	Mean (SD)	n	Mean (SD)	F (df)	η_p^2	p
Job decision authority[1]	40	3.03 (0.92)	122	3.03 (0.76)	53	2.95 (0.92)	(2, 212) = 0.20	0.002	.818
Quantitative job demands[2]	40	3.67 (0.95)	123	3.56 (0.93)	55	3.39 (0.80)	(2, 215) = 1.19	0.011	.306
Cooperation between occupational groups[3]	40	3.73 (0.89)	123	3.44 (0.81)	55	3.61 (0.86)	(2, 215) = 2.05	0.019	.132
Psychosocial Safety Climate[4]	42	24.33 (8.29)	135	28.47 (9.69)	54	28.02 (8.89)	(2, 228) = 3.26	0.028	.040*
Irritation Scale[5]	43	27.88 (11.12)	136	26.04 (10.45)	57	27.86 (9.94)	(2, 233) = 0.88	0.007	.416
Effort-Reward Imbalance Scale[6]	39	1.43 (0.36)	120	1.58 (0.34)	49	1.42 (0.40)	(2, 205) = 4.52	0.042	.012*
Job Satisfaction[7]	40	29.44 (5.14)	123	28.37 (4.83)	55	29.30 (4.62)	(2, 215) = 1.12	0.010	.328
Intention to Leave[8]	38	1.68 (0.99)	121	1.95 (0.95)	55	1.80 (0.85)	(2, 211) = 1.43	0.013	.242
Employer Attractiveness[9]	40	20.20 (6.99)	123	21.01 (6.48)	55	21.92 (6.12)	(2, 215) = 0.83	0.008	.437
Recommendation[10]	40	6.83 (2.87)	123	6.31 (2.67)	53	6.45 (2.58)	(2, 213) = 0.56	0.005	.573

[1] [37], [2] [37], [3] [37], [4] [39], [5] [43], [6] [44], [7] [45], [8] [48], [9] [49], [10] [50]. Numbers in brackets refer to the corresponding sources in the reference list.

ANOVA = analysis of variance. SD = standard deviation, df = degrees of freedom, η_p^2 = partial eta squared. *Statistically significant at $p < .05$.

Table 7s. Hochberg's GT2 post hoc comparisons between professional groups among employees without leadership responsibility.

**Outcomes**	Professional Groups	Professional Groups	Mean Difference [95% CI]	SD	Cohen's d	p
Psychosocial Safety Climate[1]	Physicians	Nurses	-4.14 [-8.08; -0.19]	1.64	-0.441	.036*
	Nurses	Others	0.46 [-3.13; 4.05]	1.49	0.048	.986
	Others	Physicians	3.68 [-0.91; 8.27]	1.91	0.427	.155
Effort-Reward Imbalance Scale[2]	Physicians	Nurses	-0.15 [-0.31; 0.01]	0.07	-0.434	.072
	Nurses	Others	0.15 [0.01; 0.30]	0.06	0.428	.038*
	Others	Physicians	-0.00 [-0.19; 0.18]	0.08	-0.010	1.000

[1] [39], [2] [44]. Numbers in brackets refer to the corresponding sources in the reference list.

CI = confidence interval, SD = standard deviation. *Statistically significant at $p < .05$.

Table 8s. Working conditions, job stress and work-related consequences: comparisons between groups with or without full-time contracts among employees without leadership responsibility. ANOVAs with effect sizes.

	Full-time working		Part-time working		Group effect		
**Outcomes**	n	Mean (SD)	n	Mean (SD)	F (df)	η_p^2	p
Job decision authority[1]	117	3.09 (0.81)	103	2.95 (0.85)	(1, 218) = 1.73	0.008	.190
Quantitative job demands[2]	118	3.58 (0.96)	105	3.52 (0.86)	(1, 221) = 0.22	0.001	.638
Cooperation between occupational groups[3]	118	3.62 (0.89)	105	3.44 (0.78)	(1, 221) = 2.57	0.011	.111
Psychosocial Safety Climate[4]	128	29.00 (10.55)	107	25.90 (7.29)	(1, 225.38) = 7.04	0.028	.009*
Irritation Scale[5]	130	25.72 (10.41)	111	28.27 (10.40)	(1,239) = 3.61	0.015	.059
Effort-Reward Imbalance Scale[6]	115	1.50 (0.37)	98	1.52 (0.37)	(1, 211) = 0.27	0.001	.604
Job Satisfaction[7]	118	29.15 (4.83)	105	28.47 (4.78)	(1, 221) = 1.12	0.005	.290
Intention to Leave[8]	116	1.84 (0.98)	103	1.92 (0.88)	(1, 217) = 0.431	0.002	.512
Employer Attractiveness[9]	118	21.57 (6.52)	105	20.57 (6.51)	(1, 221) = 1.31	0.006	.254
Recommendation[10]	118	6.70 (2.67)	104	6.18 (2.71)	(1, 220) = 2.08	0.009	.151

[1] [37], [2] [37], [3] [37], [4] [39], [5] [43], [6] [44], [7] [45], [8] [48], [9] [49], [10] [50]. Numbers in brackets refer to the corresponding sources in the reference list.

ANOVA = analysis of variance. SD = standard deviation, df = degrees of freedom, η_p^2 = partial eta squared. *Statistically significant at $p < .05$.
